# Whole genome characterization of non-tissue culture adapted HRSV strains in severely infected children

**DOI:** 10.1186/1743-422X-8-372

**Published:** 2011-07-28

**Authors:** Rajni Kumaria, Laxmi Ravi Iyer, Martin L Hibberd, Eric AF Simões, Richard J Sugrue

**Affiliations:** 1 Singapore-MIT Alliance for Research and Technology, Centre for Life Sciences, #05-06M, 28 Medical Drive, 117456, Singapore; 2 Division of Molecular and Cell biology, School of Biological Sciences, Nanyang Technological University, 60 Nanyang Drive, 639798, Singapore; 3 Genome Institute of Singapore, #02-01, Genome Building, 60 Biopolis Street, 138672, Singapore; 4 University of Colorado, Denver and The Division of Infectious Diseases, The Children's Hospital, 13123 East 16th Avenue, Aurora, CO 80045, USA

**Keywords:** Human respiratory syncytial virus, HRSV A type severe clinical strains, Whole genome sequence, Phylogenetic analysis, Protein analysis

## Abstract

**Background:**

Human respiratory syncytial virus (HRSV) is the most important virus causing lower respiratory infection in young children. The complete genetic characterization of RSV clinical strains is a prerequisite for understanding HRSV infection in the clinical context. Current information about the genetic structure of the HRSV genome has largely been obtained using tissue culture adapted viruses. During tissue culture adaptation genetic changes can be introduced into the virus genome, which may obscure subtle variations in the genetic structure of different RSV strains.

**Methods:**

In this study we describe a novel Sanger sequencing strategy which allowed the complete genetic characterisation of 14 clinical HRSV strains. The viruses were sequenced directly in the nasal washes of severely hospitalized children, and without prior passage of the viruses in tissue culture.

**Results:**

The analysis of nucleotide sequences suggested that vRNA length is a variable factor among primary strains, while the phylogenetic analysis suggests selective pressure for change. The G gene showed the greatest sequence variation (2-6.4%), while small hydrophobic protein and matrix genes were completely conserved across all clinical strains studied. A number of sequence changes in the F, L, M2-1 and M2-2 genes were observed that have not been described in laboratory isolates. The gene junction regions showed more sequence variability, and in particular the intergenic regions showed a highest level of sequence variation. Although the clinical strains grew slower than the HRSVA2 virus isolate in tissue culture, the HRSVA2 isolate and clinical strains formed similar virus structures such as virus filaments and inclusion bodies in infected cells; supporting the clinical relevance of these virus structures.

**Conclusion:**

This is the first report to describe the complete genetic characterization of HRSV clinical strains that have been sequenced directly from clinical material. The presence of novel substitutions and deletions in the vRNA of clinical strains emphasize the importance of genomic characterization of non-tissue culture adapted primary strains.

## Introduction

Human respiratory syncytial virus (HRSV) is responsible for approximately 64 million infections and 160,000 deaths each year [1]. It is the most important cause of lower respiratory tract (LRT) infection in young children and neonates, and giving rise to a spectrum of symptoms; from relatively mild to severe. Prior exposure to HRSV does not give complete protective immunity; re-infection occurs throughout life. Although HRSV infection is a major health concern in developed countries, it is a significant cause of ALRI-associated death in young children in developing countries [2]. There is currently no available vaccine, and the availability of specific antiviral drugs is limited.

The mature HRSV particle comprises a ribonucleoprotein (RNP) complex, formed by the interaction between the viral genomic RNA (vRNA), the nucleocapsid (N) protein, the phospo (P) protein, and the large (L) protein. The vRNA consists of ten contiguous genes, and each gene begins with a short non-coding region gene start (GS) sequence and ends with a gene end (GE) sequence. The first nine virus genes are separated by an additional coding sequence called the intergenic region, and the vRNA is flanked by a leader region at the 3' end, and a trailer region at the 5' end of the vRNA. Although the minimal functional polymerase activity requires an association between the N, P and L proteins and the virus genome vRNA, additional viral proteins called the M2-1 protein, M2-2 and M protein regulate the activity of the polymerase [3-10]. The virus is surrounded by a lipid envelope in which the three virus integral membrane proteins are inserted. The G protein mediates attachment of the virus to the cell during virus entry [11], the fusion (F) protein [12] mediates the fusion of the virus and host cell membranes during virus entry, while the role of the SH protein is currently unknown. In addition, two non-structural proteins called NS1 and NS2 are expressed, which are thought not to be present in the virus particle but play a role in countering the host innate immune response [13]. On the basis of antigenic differences primarily in the G gene, HRSV is divided into two main subtypes; HRSV A and HRSV B [14], which can be further subdivided into distinct lineages and genotypes based on the genetic diversity in G gene [15-18].

Molecular epidemiological studies of G gene suggest HRSVA predominates in most epidemics [17,19-24], but the association between HRSV subtype and severity of infection is uncertain The availability of complete genomic sequence information from HRSV field isolates is a prerequisite to understand the clinical basis of disease, and to better understand the biology of the virus in the clinical scenario. Currently, complete genome sequences are available only for four HRSVA strains (A2; GenBank accession number M74568, RSS-2; NC_001803, Long strain; AY911262 and Line 19; FJ614813) [25-30]. Moreover, these viral strains have been passaged in cell culture prior to genetic characterisation, which can lead to subtle genetic changes in the vRNA as a result of tissue culture adaptations. In this study, we present the in-depth analysis of whole genome sequence of 14 HRSVA primary clinical strains. These viruses were sequenced directly from clinical material that was obtained from HRSV-infected patients, and without any prior passage of the viruses in tissue culture. This study provides the first detailed genome wide comparison of primary strains with the four cultured reference strains and reveals the rare and some new substitutions found exclusively in the primary strains only in F, L, M2-1 and M2-2 genes.

## Methods

### Clinical Setting and Specimen Collection

We conducted a prospective study of previously healthy term infants less than 1 year of age admitted to The Children's Hospital, Denver, Colorado, USA during three winter seasons; year 2003-2004, 2004-2005, and 2005-2006. Nasopharyngeal washings were collected from infants who were ≤ 1 year of age at the time of enrolment into the study and screened for HRSV infection. Parents or legal guardian of the subject voluntarily signed informed consent. Patients having prior LRTI or documented wheezing disease; prior known RSV disease; diagnosis of BPD/CLD; congenital heart disease (except children with previous uncomplicated acyanotic CHD, e.g., PDA, small septal defect, who are anatomically and hemodynamically normal at the time of enrolment); mechanical ventilation (including CPAP) in the prior 6 months and known immunodeficiency were not included in this study. Ethical clearance for the study was obtained from the COMIRB of the University of Colorado, Denver.

### cDNA synthesis and PCR of complete RSVA genome

Viral RNAs was extracted from 250µl of the nasopharyngeal washings using the Trizol LS reagent (Invitrogen Life Technologies, USA), according to the manufacturer's instructions. A full length cDNA was synthesised using reverse primer P-15R (nucleotide position; nt 15198-15222), based on reference strain HRSV A2 genome sequence. Briefly, 5 µl of RNA was mixed with 1µl of 10 mM dNTPs, 1 µl of 20 µM reverse primer P-15R and 3 µl RNase free water. Mixture was heated to 65°C for 5 minutes and incubated on ice for 1 min followed by addition of 4µl of 5X RT buffer, 200U of Superscript III reverse transcriptase (Invitrogen, USA), 40U of RNase-OUT RNase Inhibitor (Invitrogen, USA), 2 µl of 25 mM MgCl_2 _and 2 µl of 0.1 mM DTT. The reaction was incubated at 50°C for 50 min and then heated at 85°C for 5 min to terminate the reaction. 1 µl of RNase H was added per reaction tube and incubated at 37°C for 20 min.

The viral genome was amplified as 15 overlapping PCR fragments covering the full length of this cDNA. Each of forward primer had M13/pUC (-20) primer and reverse primer having M13R-pUC(-26) primer sequence incorporated at its 5' end (Additional file 1 Table S1). Briefly, 1 µl cDNA was added to PCR mixture containing 39.75 µl of distilled water, 5µl of 10X PCR buffer, 1.25µl of 10 mM dNTPs, 1 µl each of 20 µM forward and reverse primer and 1U of *pfu *Ultra II fusion HS DNA polymerase (Stratagene, USA). Initial denaturation at 95°C for 1 min was followed by 40 cycles of PCR with each cycle of denaturation for 20 sec at 95°C, annealing for 20 sec at 55°C and elongation for 45 sec at 72°C, with a final extension cycle of 5 min at 72°C. The PCR products were separated by electrophoresis using 1% agarose gel and visualized using 1X GelRed (Biotium, CA).

### DNA sequencing

PCR products were purified and nucleotide sequencing was performed on both forward and reverse strands of each fragment using ABI Big Dye Terminator v3.1 Reaction kit (Applied Biosystems, USA) and analysed using ABI 3730XL DNA Sequencer (Applied Biosystems, USA). As in every PCR product, the 5' end of forward strand had M13/pUC (-20) primer sequence and the 5'end of its reverse strand had M13R-pUC(-26) primer sequence, thus sequencing of the forward and reverse strands of all the PCR fragments was carried out using M13/pUC (-20) primer and M13R-pUC(-26) primer respectively. In addition, a set of internal forward and reverse sequencing primers were designed and used for each fragment to obtain complete 2 fold sequencing coverage (primer sequences available on request). The 3' end terminal region of genome was sequenced with help of reverse primer RSVSTART- (primer binding position nt 341- 322, which is in the NS1 gene), which moves towards 3' end of genome till nucleotide 1 (start of genome). While the 5' end of the genome was sequenced with help of primer RSVEND (primer binding position nt 14917-14938, in the L gene), which covered the sequence till 5' end of the genome. (primer sequences available on request). The strategy of amplification and sequencing was first standardized using RSVA2 lab strain before its adaptation for clinical strains. The fourteen HRSVA genome sequences have been deposited into GenBank [Accession numbers GU591758- GU591771].

### Phylogenetic analysis

The nucleotide sequences were mapped to HRSVA2 reference strain using Seqscape software 2.5 (Applied Biosystems, USA) and resultant consensus sequences were used for phylogenetic analysis using MEGA 4.0.2 and CLUSTAL W software. The published complete nucleotide sequences of all the four cultured HRSV A strains: A2 (GenBank accession numbers M74568), RSS-2 (NC_001803), Long (AY911262), Line 19 (FJ614813) and one HRSV B reference strain 9320 (AY353550) were downloaded from NCBI GenBank for comparison. Phylogenetic tree was estimated by Neighbor-joining (NJ) method[31]. The evolutionary distances were computed using the Maximum Composite Likelihood method [32]. The statistical robustness and reliability of the branching order within the phylogenetic tree were confirmed through a bootstrap analysis using 1,000 replicates for the NJ tree [32,33]. EMBOSS Transeq (EMBOSS EBI), an online bioinformatics translation tool was used for translation of the nucleotide sequences of genes to protein sequences.

### Immunofluorescence microscopy

Cells were labeled as described previously [34]. Briefly, cells on 13 mm glass cover slips were generally fixed with 3% paraformaldehyde in PBS for 30 min at 4°C, permeabilised using 0.1% saponin, and then labeled with primary antibodies anti-RSV (Novocastra; Anti-RSV composite antibody preparation, which recognizes the N, P, M2-1 and F proteins,) and a secondary antibody conjugated to FITC. The stained cells were mounted on slides using Citifluor™ and visualized using a Nikon eclipse 80i fluorescence microscope.

## Results and Discussion

### 1. Strategy developed for HRSVA whole genome amplification and nucleotide sequencing

Although complete genomic sequence has been reported for RSVA2, RSS, long and Line 19 virus isolates [25,27-30], the viruses were passaged in tissue culture prior to sequencing the vRNA. In this current report we present the first complete genetic characterisation of HRSVA clinical strains, obtained directly from patient specimens without prior passage of the viruses in tissue culture. This avoided the acquisition of genetic changes due to tissue culture adaptation, and enabled us to detect subtle sequence variations in the vRNA of these viruses, changes that could in principle be confused with genetic changes that arise from tissue culture adaptations. We adopted a simple approach for sequencing of vRNA in clinical specimens that circumvented the need for prior growth in tissue culture. A major advantage of this methodology is that complete vRNA sequence information can be obtained from clinical specimens even when the vRNA is low (e.g due to low viral load), and in cases where the virus strains can't be recovered even by growth in tissue culture.

**Figure 1:**
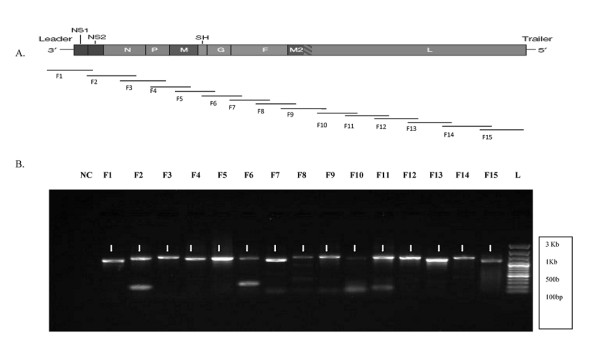
**Schematic representation of HRSVA whole genome amplification of HRSV-1 by Reverse transcriptase (RT)-PCR**. A. The viral genes with leader at 5' end and trailer at 3' end are schematically presented in the 15,222 bases HRSVA2 genome. The thin lines given below represents the approximate size of each of the fifteen amplified fragments (F) and the position of the respective fragments on the genome. The lines are to the approximate scale. B. All the PCR amplified fragments were run by electrophoresis on 1% agarose gel in TAE buffer and visualized by gel red. The fragments are numbered from F1-F15 and their positions on gel are indicated by arrows. Negative control (NC) is the PCR reaction with water in place of sample. The DNA ladder (L) has highest band position at 3 Kbp and lowest band at 100 bp. The size of fifteen fragments is as F 1 = 1215 bp, F2 = 1343 bp, F3 = 1417 bp, F4 = 1301 bp, F5 = 1373 bp, F6 = 1328 bp, F7 = 1324 bp, F8 = 1392 bp, F9 = 1435 bp, F10 = 1328 bp, F11 = 1403 bp, F12 = 1360 bp, F13 = 1200 bp, F14 = 1334 bp, F15 = 1168 bp.

The genome wide sequence analysis was conducted on fourteen primary HRSVA strains isolated from hospitalized children with severe HRSV infection (referred to as RSV-1 to RSV-14). The strategy for the nucleotide sequencing of the vRNA generated 15 overlapping PCR products, which covered the entire virus genome length This experimental strategy enabled us to produce high concentration of specific DNA products for all the fifteen fragments from each of the clinical strains. (Figure 1A). A full genome length single stranded cDNA representing the vRNA was synthesized by using reverse primer (P-15R) that binds at the 5'end of the vRNA till the last nucleotide of genome. This cDNA was sufficient for formation of all the fifteen PCR fragments using fragment specific primers (Figure 1B). As the first forward primer (P-1F) binds to the first nucleotide of the genome and the fifteenth reverse primer (P-15R) binds till the last nucleotide at the 5'end of the vRNA, the full genome was amplified with help of fifteen primer sets. Each fragment was designed to have approximately 300 base pair overlap with adjacent fragments for better genome sequence coverage. Presence of M13 forward and reverse primer sequence in each PCR product helped the sequencing of all fifteen fragments in both the forward and reverse directions using these two primers only. Internal sequencing primers were used to sequence small sequencing gaps following sequencing of PCR fragments. The 3' end of genome could be completely sequenced using the reverse orientation primer RSVSTART and likewise 5' end was sequenced using a forward orientation primer RSVEND nucleotide position are with reference to A2 strain). RSVSTART binds to region in NS1 gene and sequence in reverse direction to cover till nucleotide 1 (start of genome), RSVEND primer binds in the L gene and covers the genome sequence till last nucleotide of genome. The methodology was initially evaluated using the well characterised HRSV A2 laboratory strain, and thereafter successfully applied to fourteen clinical strains.

### 2. Primary clinical strains: Phylogenetic analysis and characterization of viral genome

**Figure 2:**
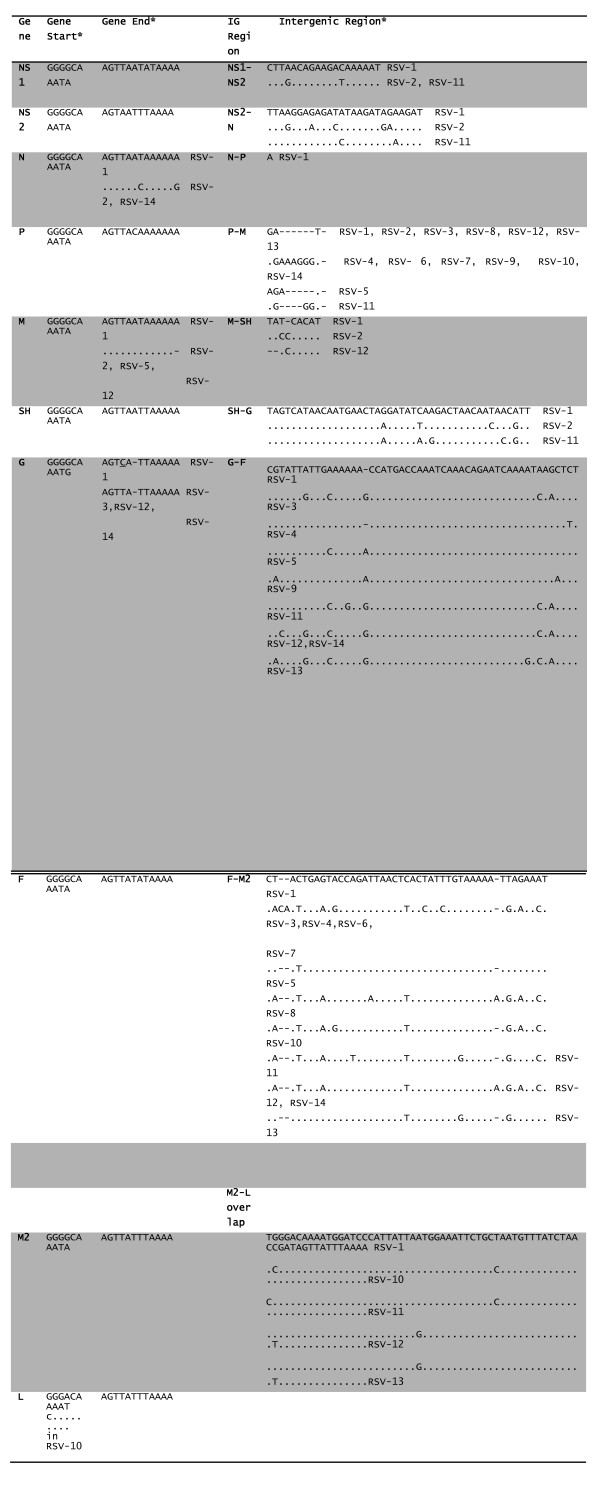
**Nucleotide alignment and comparative analysis of the Gene Junction (Gene Start - Intergenic Region -Gene End) in strains from hospitalized patients**. Genes identification is denoted before their corresponding GS and GE. Similarly corresponding intergenic region positions have also been denoted. An overlap instead of intergenic region is present between M2-L genes. *Clinical strains having difference in the sequences are shown in the table. Thus rest of the sequences matched RSV-1.

The length of the vRNA from the clinical strains ranged from 15,210 to 15222 nucleotides, with RSV-2 and RSV-7 having the shortest and longest vRNA respectively (Table 1). The viral genome length of primary isolates is variable, similar to that reported in the prototype strains [25-30], with only one clinical strain (RSV-7) having the same size vRNA as the RSV A2 isolate. The variations were attributed mainly to deletions observed in the non-translated regions, mainly between P-M and F-M2 (Figure 2).

**Table 1:** Comparison of the genome length (nucleotides) of the fourteen HRSVA clinical strains and transition bias (R) in clinical strains with reference to strain RSV-1 (Clinical reference strain)

RSV A	Genome Length	% Nucleotide Variability	Transition Bias (R)
RSV -1^1^	15,212	----	Ref strain (RSV-1)

RSV-2	15,210	1.42	2.4

RSV-3	15,218	2.36	2.3

RSV-4	15,219	2.14	2.4

RSV-5	15,214	0.6	2.1

RSV-6	15,220	1.72	1.2

RSV-7	15,222	1.42	1.4

RSV-8	15,212	1.95	1.9

RSV-9	15,219	1.15	1.8

RSV-10	15,218	2.38	2.2

RSV-11	15,215	3.3	2.1

RSV-12	15,211	3.1	2.8

RSV-13	15,215	2.9	2.7

RSV-14	15,218	1.88	1.9

**Lab adapted reference strain**			

RSV A2	15222	3.30	2.9

RSS	15191	2.67	2.4

Long	15226	3.56	2.9

Line 19*	15191	3.58	2.9

^1^RSVA2 (lab adapted strain) is passaged several times in cell culture, thus may have numerous culture mediated mutations. Therefore, RSV-1, a strain obtained from a hospitalized RSVA patient is used as a reference strain for comparative analysis of RSVA strains from clinical source and published reference strains. * Nucleotide sequence for 5' and 3' ends of Line 19 is not available.

**Figure 3:**
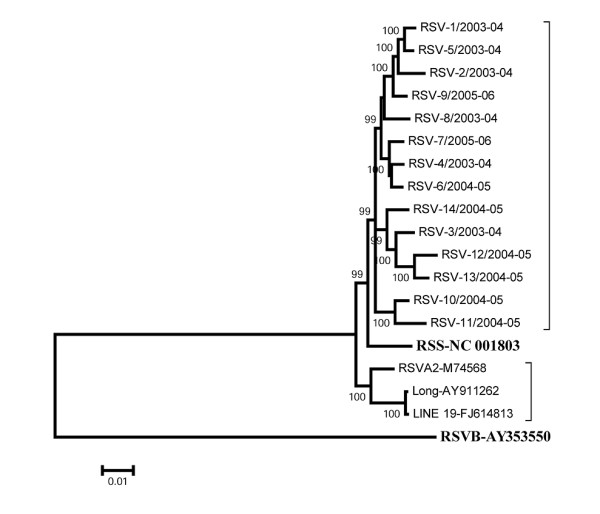
**Phylogenetic relationship of 14 clinical strains of HRSVA from patients and 4 representative strains from the Genbank based on whole genome nucleotide sequence**. Hospitalized patient strains have been indicated with prefix RSV. The reference strains are indicated by their Genbank accession number. The evolutionary history was inferred using the Neighbour-Joining method. The bootstrap values below 80% are not shown. The scale bar indicates 1% nucleotide sequence divergence. The HRSVB type (Accession number AY353550) has been used as the root.

We observed a generally high level of sequence conservation among the clinical strains examined in this study, and between the clinical strains and several RSV prototype strains. This suggested that selection pressure is towards conservation and/or that the genomic structure of HRSV may be relatively constrained. Phylogenetic analysis showed that the clinical strains represented a distinct lineage within HRSV A group, separate from the previously published cultured strains (Figure 3). The transition bias (R), which is an important parameter of genetic sequence evolution, ranged between 1.2 and 2.8 for the clinical strains, also suggested comparatively lower evolution selection rate.

### 3. Comparison of protein sequences encoded by the clinical strains with prototype and published strains

All genetic sequences from the clinical and prototype strains were translated *in silico*, and the resulting predicted protein sequences were compared.

#### 3.1 Integral membrane proteins

The clinical strains showed a higher sequence variation in the G protein, which is consistent with a recent study suggesting selection pressure on G protein based on Bayesian method implemented in the HyPhy program [35]. The clinical strains exhibited between 2.0 and 6.4% variation in the amino acid sequence, and this variation was located mainly in the ectodomain region (Table 2, Additional file 2 Figure S1). Bio-informatics analysis has identified 29 amino acid positions in the G protein that are under positive selection pressure [35]. Comparison of the G protein sequence in the 14 clinical strains indicated the presence of 14 of these sites, at L-215-P, P-222-S, V-225-A, P-226-F, T-227-S, P-256-L, F-265-L,S-269-T, T-274-P, I-279-V, L-286-P, P-289-S, P-290-L, S-293-P. Amino acid variation observed at five of these sites (position 274, 279, 286, 290 and 293) differed in the most of the clinical strains compared to the four cultured reference strains (Table 3). Antigenic epitopes have been identified in the G protein [36-38] and we noted a degree of amino acid sequence variation at these sites. The following amino acid changes in the G protein of the clinical strains; T-244-R (in RSV-2, RSV-13, ) and F-265-L (in RSV-2, RSV-3, RSV-5, RSV-9, RSV-11, RSV-13, RSV-14) may be associated with loss of these antigenic epitopes.

**Table 2:** Amino acid variability (%) identified in individual proteins of HRSVA: Comparison between hospitalized strains

Strain	HRSVA Proteins (% Amino acid variability)										
	**NS1**	**NS2**	**N**	**P**	**M**	**SH**	**G**	**F**	**L**	**M2-1**	**M2-2**

RSV-1^1^											

RSV-2	0.7	2.4	0.25	0	0	0	5.4	1.21	0.27	0.25	0

RSV-3	0	0	0.25	0.4	0	0	5.4	1.74	1.06	0.33	0.88

RSV-4	0	0	0.5	0	0	0	2.34	1.91	1.01	0.33	0.44

RSV-5	0	0	0	0.4	0	0	3.3	1.91	0.18	0.25	0

RSV-6	0	0	0	0	0	0	2.7	1.91	0.69	0.33	0.55

RSV-7	0	0	0.5	0	0	0	2.7	1.74	0.2	0.33	0.44

RSV-8	0	0	0	0	0	0	2.01	1.39	0.46	0.25	0

RSV-9	0	0	0	0	0	0	2.34	1.21	0.54	0.38	0

RSV-10	0	0	0.25	0.4	0	0	2.01	1.56	1.08	0.38	0.33

RSV-11	0.7	2.4	0.5	0	0	0	6.4	2.09	1.15	0.38	0.44

RSV-12	0	0	0.5	0.4	0	0	5.4	1.91	1.38	0.55	0.55

RSV-13	0	0	0.5	0.4	0	0	6.4	2.61	1.47	0.60	0.55

RSV-14	0	0	0.5	0	0	0	5.7	2.61	0.55	0.55	0.44

HRSVA2 (lab adapted strain) is passaged several times in cell culture, thus may have numerous culture mediated mutations. Therefore, RSV-1, a strain obtained from a hospitalized RSVA patient is used as a reference strain for comparative analysis of the proteins of RSVA strains from clinical source.

**Table 3:** Comparative analysis of G protein amino acid positions under positive selection pressure in primary strains compared with prototype strains

Amino acid	Prototype Strains				Primary Clinical
215L		RSS	LONG	L-19	RSV-1, RSV-2

215P	A2				RSV-3 to RSV-14

222P		RSS	LONG	L-19	RSV-1, RSV-2, RSV-11

222S	A2				RSV-3 to RSV-10, RSV-12 to RSV-14

225V	A2	RSS	LONG	L-19	RSV-1, RSV-3 to RSV-10

225A					RSV-2, RSV-11

226P	A2	RSS	LONG	L-19	RSV-1 to RSV-10,RSV-12 to RSV-14

226F					RSV-11

227T	A2	RSS	LONG	L-19	RSV-1, RSV-3 to RSV-14

227S					RSV-2

256P	A2	RSS	LONG	L-19	RSV-1, RSV-3, RSV-4, RSV-6 to RSV-10, RSV-12, RSV-14

256L					RSV-2, RSV-5, RSV-11

256Q					RSV-13

265F	A2		LONG	L-19	RSV-1, RSV-4, RSV-6 to RSV-8, RSV-10, RSV-12

265L		RSS			RSV-2, RSV-3, RSV-5, RSV-9, RSV-11, RSV-13, RSV-14

269S	A2	RSS	LONG	L-19	RSV-1, RSV-2, RSV-4 to RSV-12

269T					RSV-3, RSV-13, RSV-14

274T					RSV-1, RSV-2, RSV-4 to RSV-6, RSV-8 to RSV-10

274P	A2	RSS			RSV-3, RSV-11 to RSV-14

274L			LONG	L-19	

274S					RSV-7

279I					RSV-1 to RSV-10

279V	A2	RSS	LONG	L-19	RSV-11 to RSV-14

286L		RSS			RSV-1 to RSV-10

286P	A2		LONG	L-19	RSV-11

289P	A2	RSS	LONG	L-19	RSV-1, RSV-2

289S					RSV-3, RSV-12 to RSV-14

290P		RSS			RSV-1, RSV-2, RSV-4 to RSV-11

290L					RSV-3, RSV-12 to RSV-14

290S	A2		LONG	L-19	

293S		RSS			RSV-1 to RSV-14(All clinical strains)

293P	A2		LONG	L-19	

The G protein plays an important role in attachment of the virus to the host cell [11], along with several cellular factors which have been proposed to mediate HRSV attachment [39,40]. At least two interactions between the G protein and cellular factors have been described, and the properties of the G proteins that mediate these interactions are conserved in the clinical strains. The G protein, amino acid motif 182-186, which is proposed to have structural similarity to CX3C chemokine fractalkine [40], is completely conserved in all the primary strains. Similarly the heparin binding site and the cysteine rich central domain in the G protein [41] were completely conserved in all the clinical strains.

The F protein exhibited a relatively higher sequence conservation [Additional file 3 Figure S2], which presumably reflects its importance in mediating virus entry, and sequence variation was highest in the signal sequence, transmembrane and cytoplasmic domains. The essential features of the F protein were largely conserved in the clinical strains, including the two furin cleavage sites and the potential N-linked glycosylation sites. These furin cleavage sites have been proposed to generate a short 27 amino acid glycopeptide in virus-infected cells [42], and among the clinical strains we observed a relatively high degree of sequence variation in the putative glycopeptides. The biological significance of this in humans is currently unclear, although the corresponding glycopeptide in the closely related bovine RSV exhibits tachykinin activity [43]. Several neutralizing antibody epitope sites have been identified, including 7C2 (aa221-236), 47F (aa262-268) and RS-348 (aa205-225) [44] and these are completely conserved in all clinical strains. Of sequences related to cytotoxic T-lymphocyte (CTL) epitopes, a single substitution was observed at F-547-L, which has been reported in the HLA Cw*12 CTL epitope [45].

The recently characterized cell cultured Line 19 strain is suggest to be highly mucogenic [28], and is found to have six unique amino acid differences in the F protein when compared with the F protein sequence in HRSV A2 and long type [28]. However, neither of these differences were observed in the F protein sequence of the clinical strains, thus the clinical relevance of these F protein sites is uncertain. Palivizumab (PZ) is the humanized murine monoclonal Ab (mAb) widely used for prophylaxis against RSV infection in high risk infants and children that binds to the F protein at aa422-438 [46]. A panel of resistant mutations in the F gene in the binding site, F212, MP4, MS312, MS412 MS512 have been identified against PZ *in vitro *and *in vivo *studies [47-49]. Also, a number of mutations have been identified in the coding regions for the binding sites on the F protein for MAb19, RHZ19 and ch101F, other potent mAbs, with clinical potential [49,50]. There were no sequence variation at these sites in the clinical strains examined in our study.

The SH membrane protein was completely conserved in all the clinical strains. While the sequence conservation of the SH has been previously reported, suggesting that it may be clinically relevant [51], and the SH protein is dispensable for virus replication in tissue culture [52]. Our own studies employing siRNA to inhibit SH gene expression (Ng and Sugrue, unpublished observations) support this observation.

#### 3.2 Polymerase associated proteins

The L, P, N and M2-1 genes which encode the polymerase and associated proteins also showed very low sequence variability. Alignment of deduced L protein sequence of our strains with other published sequences on NCBI BLAST revealed that presence of asparagine or tyrosine at position 148 and glycine at 174 are exclusive to our clinical strains The variation at aa148 led to replacement of negatively charged amino acid with that having neutral side chain and at aa174, it changed the polar negatively charged amino acid with non polar neutral. These substitutions are located before domain I of L protein, proposed as nucleotide binding domain [53,54]. There were few amino acid variations in the N, L and P proteins. Two substitutions identified in the N protein of clinical strains, L-64-V was identified in seven (RSV-1, RSV-2 RSV-3 RSV-5 RSV-6 RSV-7 RSV-9) and R-84-K in five (RSV-8, RSV-11 to RSV-14). Although the overall sequence of the L protein was comparatively conserved among the clinical strains, we observed that M-59-I, L-81-I, I-2016-V differed from that in the A2, Long and Line 19. Two exclusive differences found predominantly among our clinical strains were at D-148-N/Y and V/D-174-G in strains RSV-1, RSV-2, RSV-4 to RSV-10, RSV-14 and strains RSV-1 to RSV-9, RSV-14 respectively. (Table 4; Additional file 4 Figure S3). With the exception of a single amino acid difference at A-73-V/T in RSV-3, RSV-10, RSV-11, RSV-12 RSV-13, the P protein remained conserved in all the clinical strains. The M protein showed 100% conservation in all the clinical strains examined (Table 2).

**Table 4:** Nucleotide changes leading to amino acid substitutions in F, L, M2-1 and M2-2 proteins exclusively in primary clinical HRSVA strains as compared to reference strains

Protein	Nucleotide change in Primary strains	Amino acid substitution - Position	Primary strains having substitution
F	CTC/CTT → TTT	L-15-F	RSV-1, RSV-2,RSV-7 to RSV-10

L	GAC → AAC/TAC	D-148-N/Y	RSV-1, RSV-2, RSV-4 to
	GTC/GAC → GGC	V/D-174-G	RSV-10, RSV-14
			RSV-1 to RSV-9, RSV-14

M2-1	ATA → CTA	I-87-L	RSV-12 to RSV-14
	AAA → AGT	N-117-S	RSV-8, RSV-10, RSV-11

M2-2*	ATG → ACG	M-1-T	RSV-1, RSV-2, RSV-5, RSV-8 to RSV-14
	ACT → AAT	T-18-N	RSV-1, RSV-2, RSV-5, RSV-7 to RSV-14
	AGA → AAT/AGT	R-25-N/S	RSV-1, RSV-2, RSV-5, RSV-8 to RSV-14
	TTC → ATC	F-39-I	RSV-12 to RSV-14
	CCA → CAA	P-44-Q	RSV-1, RSV-2, RSV-4, RSV-5, RSV-8 to RSV-14
	ATG → ATA/ACG	M-48-I/T	RSV-1, RSV-2, RSV-5, RSV-7 to RSV-14
	CCA → CAG	P-51-Q	RSV-12 to RSV-14
	ACA → CCA	T-54-P	RSV-1, RSV-2, RSV-4 to RSV-14
	ACA → GCA	T-68-A	RSV-1 to RSV-14
	ATT → ACT	I-69-T	RSV-3 to RSV-10, RSV-14
	ATT → ACT	I-79-T	RSV-1 to RSV-6, RSV-8, RSV-9, RSV-12 to RSV-14
	GAG → GAT	E-80-D	RSV-1 to RSV-14

*In the deduced M2-2 protein sequence, the RSS strain had difference at only I-48-T.

The transcription elongation factor M2-1 is highly conserved among the clinical strains, along with the Cys(3)-His(1) motif that is important for its functionality [55,56]. While amino acid variability was extremely low for the M2-1 protein (between 0.25-0.6%) among clinical strains, exclusive substitutions I-87-L in RSV-12 to RSV-14 and N-117-T/S in RSV-8, RSV-10, RSV-11-14 were observed (Table 4; Additional file 5 Figure S4). M2-2 is proposed to shift the balance from vRNA transcription to vRNA replication [57,58]. We noted a degree of M2-2 protein sequence variation between the clinical strains and those reported for several HRSV prototype viruses. In addition, the absence of the first start codon in 71% of the clinical strains suggested the expression of shorter M2-2 protein.

Interestingly, the M2-2 protein sequence showed a relatively large number of sequence variations when compared with prototype cultured viruses. We observed a 11.11% difference in the M2-2 protein sequence of all the clinical strains as compared to the reference strain (Table 4). The substitution M-1-T effectively removed the first start codon, and thus M2-2 is predicted to be 88 aa rather than 90 aa in length in 10 clinical strains due to availability of second start codon for the protein (Additional file 6 Figure S5). As the M2-2 protein plays a role in vRNA replication, the functional significance of the sequence variations in the M2-2 protein in RSV vRNA replication will require further examination.

In four clinical strains nucleotide substitutions have been observed at five different positions in M2-L gene overlap sequence, while changes at only two of positions were reported earlier by Moudy et al [59]. Moreover, nucleotide change A-26-G observed in RSV-12 and RSV-13 led to changes of amino acid N-6-S in L protein.

The M genes nucleotide sequence was completely conserved in the clinical strains, which is consistent with the importance of the M protein, both as a major structural protein, and a regulator of virus polymerase activity [9]. 3.3 Non-structural proteins:

Both the NS1 and NS2 proteins were highly conserved, with only one amino acid substitution L-105-I in the NS1 protein of RSV-2, RSV-11, and three subsitutions in the NS2 protein (D-7-G, N-8-T, K-38-R) present in the same clinical strains (RSV-2, RSV-11). The NS1 and NS2 proteins showed little sequence variation, underlining the essential roles these proteins play in evading the host innate immune response [60]. Recently siRNA targeting the NS1 protein has been proposed as an effective therapeutic strategy [61], and the conserved nature of the NS1 nucleotide sequence suggests that these siRNA molecules will also be effective against a range of RSV strains in the severe clinical scenario.

### 4. Sequence analysis of the non-transcribed gene junction and extragenic regions

The non translated regions of the vRNA are likely to play important roles in the regulation of virus genes expression [62]. The nucleotide sequence of the leader region was highly conserved, with only a single A to G nucleotide substitution at nt29 in the strains RSV-2, RSV-11. All the clinical strains had Cytosine at nt4 in the leader sequence, which is important in the context of virus replication [63]. Although we noted an overall conservation in the trailer sequence, some regions of increased sequence variation were apparent. Moreover, the length of trailer region varied between 153-162 nt among the clinical strains. The sequence for the proposed polymerase binding site (nt 1-11 of leader) [64] is completely conserved among all clinical strains. While GS sequences were well conserved among all clinical strains, the GE sequences showed a higher degree of variation. The GS sequences were conserved in the first 9 genes, and only the GS sequence preceding the L gene showed some variation. A subsitution in the GS sequeunce (to U or A) at nt1 was reported to significantly reduce transcription levels [65], but the functional significance of G to C found in the GS region of L polymerase gene is uncertain (Figure 2). The GE was conserved for 8 genes. The GE sequence of the N gene had a change at nt 7 and nt 13 in RSV-2, RSV-14, the M gene had one nucleotide shorter in RSV-2, RSV-5, and RSV-12, while in 78% of clinical strains there was a substitution at nt4 in GE sequence of the G. The changes in GE sequence of the G gene such as substitution at nt4 and shorter central region have been associated with altered the transcription termination efficiency [66,67].

By comparison with the GS and GE regions, the IG regions showed a higher degree of sequence variation. The IG sequence of the P-M gene junction varied in length between 3-9 nts, while that of the G-F and the F-M2 gene junctions also exhibited greater sequence variation amongst clinical strains. Although the significance of this sequence variation among the clinical strains is uncertain, *in vitro *studies have demonstrated that the IG region can play important role in regulating virus gene transcription [68]. It is therefore possible that these sequence variations may lead to altered viral gene expression characteristics among the different clinical strains.

### 4. Biological properties of the clinical isolates

**Figure 4:**
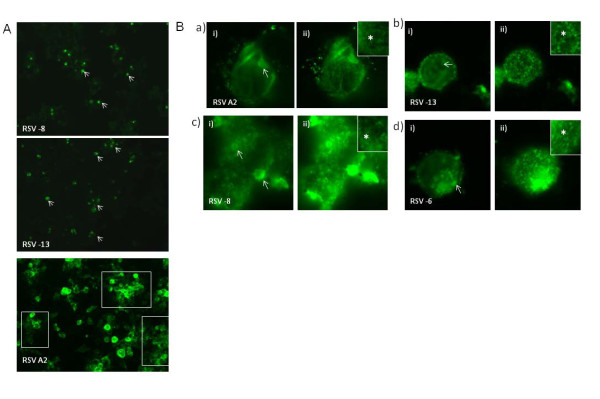
**Immunofluorescence examination of cells infected with the HRSV clinical strains**. **(A) **Differential infection levels were observed with clinical isolates as compared to lab strain RSVA2. More HEp 2 cells were seen infected with RSVA2, when compared with clinical isolate RSV-8 and RSV-13 between 2 and 4 days post infection. **(B)**. Clinical isolates (b) RSV-13, (c) RSV-8 and (d) RSV-6 also produced similar structures like (a) RSVA2. HEp2 cells were infected with RSVA2 and clincial isolates were stained with anti-RSV antibodies and visualized by immunofluorescence using secondary antibodies conjugated to FITC. Examination of the stained cells at a focal plane showing mainly the i) interior and ii) surface of infected cells are shown in each case. The presence of large cytoplasmic inclusion bodies highlighted by white arrow and presence of structures that resembled the VF are highlighted by star.

HRSV remains largely cell-associated, and infection occurs by direct cell to cell contact [34]. In cells infected with the prototype HRSV isolate A2, two distinct virus structures are formed during the virus replication cycle; the virus filaments (VF) and inclusion bodies (IB). The VF are membrane-bound structures that are 150-200 nm thick and can be up to 4 µm in length. They form at the sites of virus assembly and are the mature progeny viruses [34]. The inclusion bodies form in the cytoplasm of infected cells and can be several µm in diameter, consisting of RNP complexes, together with several essential cellular factors. These structures have been extensively examined using laboratory HRSV isolates (e.g. HRSV A2), and we examined HEp2 cells infected with the RSV clinical strains. Most of the RSV strains described in this study could not be recovered using tissue culture, which may be due to differences in the level of infectious virus particles in the starting clinical material. This is difficult to estimate using molecular techniques (e.g. qPCR), which only assesses the levels of vRNA copies, and does not distinguish between infectious and non-infectious virions. However, we were able to recover three clinical strains; RSV-4, RSV-8 and RSV-13 from infected HEp2 cells. This was confirmed by labeling of cells infected with the clinical strains using the anti-RSV and anti-mouse IgG conjugated to FITC as described previously [34], which allows visualisation of both the virus filaments and inclusion bodies. In each case infection with the clinical isolates could only be detected between 2 and 4 days post infection, and the stained cells appeared either singly or in small clusters (Figure 4A; white arrowhead). This was considerably slower compared to HRSV A2 (Figure 4A), where large numbers of infected cells and extensive syncytia could be detected by day 2 (Figure 4A; highlighted by white box). This is consistent with recent observations suggesting slower replication kinetics of clinical HRSV strains compared to laboratory prototype isolates [69] Examination of the stained cells revealed the presence of large cytoplasmic inclusion bodies (Figure 4B; highlighted by white arrow), as well as structures that resembled the VF (Figure 4B; highlighted by *). This suggests that the clinical strains produce structures similar to that observed in HRSV A2 infected cells. Several cellular factors have been identified within inclusion bodies and virus filaments using RSV prototype strains, and these cellular factors have been implicated in aspects of the HRSV replication cycle e.g. virus particle assembly [70]. The formation of similar structures in cells infected with the clinical strains suggests a similar mechanism in both HRSV A2 and the clinical strains during virus replication, and supports a clinical role these structures during HRSV infection.

## Conclusions

We report the complete genetic characterisation of fourteen clinical HRSV strains that were sequenced directly from clinical material obtained from severely ill children. In general a high degree of nucleotide sequence conservation was observed, both between the different clinical strains, and between the clinical and prototype strains. This was consistent with a low evolution rate for HRSV. Analysis of the protein coding regions of the HRSV genomes indicated that the G protein showed the greatest sequence variation between the clinical stains. Although the F protein showed a small degree of sequence variation, the essential features of the F protein (e.g. protease cleavage site) were conserved, together with several important antigenic epitopes. The protein coding region of the M and SH genes were entirely conserved, while all other virus genes showed small degrees of sequence variation. In some clinical strains the M2-2 gene showed an alternative translational start site, which would be expected to give rise to a smaller M2-2 protein lacking the first two amino acids. Analysis of the non-translated regions between the clinical strains indicated that leader and trailer regions region were highly conserved, although a small degree of sequence variation at specific regions in the trailer region was noted. The gene start regions showed a high degree of sequence conservation, while the gene end sequences were conserved for 8 genes. In contrast the intergenic regions showed a significantly higher degree of sequence variation between the different clinical strains. In tissue culture cells the clinical strains grew much slower than the prototype HRSV A2 stain. However, the formation of inclusion bodies and virus filaments were observed in HEp 2 cells infected with either the prototype A2 stain or clinical strains, suggesting a clinical relevance for these virus-induced structures.

## Abbreviations

HRSV: Human respiratory syncytial virus; NT: Nucleotides; VF: Virus filaments; IB: Inclusion bodies; LRTI: Lower Respiratory Tract infections; BPD: Bronchopulmonary dysplasia; CLD: Chronic Lung Disease; CHD: Coronary Heart Disease; PDA: Patent Ductus Arteriosus; CPAP: Continuous Positive Airway Pressure; DTT: Dithiothritol; RT: Reverse transcriptase; FITC: fluorescent isothiocyanate; GS: Gene start; GE: Gene end; IG: Intergenic region; PZ: Palivizumab; mAb: monoclonal antibodies; ALRI: acute lower respiratory infections; PBS: Phosphate Buffered Saline.

## Competing interests

The authors declare that they have no competing interests.

## Authors' contributions

RK performed the sequencing analysis and assisted in drafting the manuscript, LIR performed virus tissue culture experiments and assisted in formatting of the manuscript. MLH, EAFS and RJS conceived of the study and participated in its design and coordination. All authors have read and approved the final manuscript.

## Supplementary Material

Additional file 1 **Table S1: **Oligonucleotide primers used for reverse transcriptase-polymerase chain reaction (RT-PCR) amplification of HRSVA clinical strains and HRSVA2 strains Click here for file

Additional file 2 **Figure S1: **Amino acid sequence alignment and comparative analysis of glycoprotein between primary HRSVA strains and prototype cultured strains. The domain name with amino acid position is indicated above the sequence alignment. Click here for file

Additional file 3 **Figure S2: **Amino acid sequence alignment and comparative analysis of fusion protein between primary HRSVA strains and prototype cultured strains. The domain name with amino acid position is indicated above the sequence alignment. All the glycosylation sites are given in bold and underlined. Click here for file

Additional file 4 **Figure S3: **Amino acid alignment and comparative analysis of L-protein between primary HRSVA strains and prototype cultured strains. Click here for file

Additional file 5 **Figure S4: **Amino acid alignment and comparative analysis of M2-1 protein between primary HRSVA strains and prototype cultured strains. Click here for file

Additional file 6 **Figure S5: **Amino acid alignment and comparative analysis of M2-2 protein between primary HRSVA strains and prototype cultured strains. Click here for file
