# Supplementary material for: Whole genome characterization of non-tissue culture adapted HRSV strains in severely infected children
Source: Virol J. 2011 Jul 28;8:372. doi: 10.1186/1743-422X-8-372 (PMC3166936; doi:10.1186/1743-422X-8-372)
Supplement: Additional file 1 — Table S1: Oligonucleotide primers used for reverse transcriptase-polymerase chain reaction (RT-PCR) amplification of HRSVA clinical strains and HRSVA2 strains [file 1743-422X-8-372-S1.DOC]

Table S1:

| PCR Primer | Sequence (5’ 3’)* | Direction | Nucleotide  Position** |
| --- | --- | --- | --- |
| **P-1F** | GTAAAACGACGGCCAGACGCGAAAAAATGCGTACAAC | Forward | 1 |
| **P-1R** | CAGGAAACAGCTATGACTGGATGGTGTATTTGCTGGA | Reverse | 1216 |
| **P-2F** | GTAAAACGACGGCCAGATTTCAACACAAKAKTCACACAA  (K=G/T) | Forward | 1003 |
| **P-2R** | CAGGAAACAGCTATGACTTCAGGAGCAAACTTTTCCAT | Reverse | 2346 |
| **P-3F** | GTAAAACGACGGCCAGAAAAATTGGGTGGWGAAGCA (W=A/T) | Forward | 2014 |
| **P-3R** | CAGGAAACAGCTATGACCCCTTGGGTGTGGATATTTG | Reverse | 3431 |
| **P-4F** | GTAAAACGACGGCCAGAACCTRTTGGAAGGGAATGA (R=A/G) | Forward | 3019 |
| **P-4R** | CAGGAAACAGCTATGACAGGCCAGAATTTGCTTGAGA | Reverse | 4320 |
| **P-5F** | GTAAAACGACGGCCAGACMAACMCTCTGTGGTTCAA (M=C/A) | Forward | 4076 |
| **P-5R** | CAGGAAACAGCTATGACCAGGAAATCCAGAACWCACAA(W=A/T) | Reverse | 5449 |
| **P-6F** | GTAAAACGACGGCCAGAAGTCAACCCTGCAATCCAC | Forward | 5024 |
| **P-6R** | CAGGAAACAGCTATGACGCATTAACACTAAATTCCCTGGT | Reverse | 6352 |
| **P-7F** | GTAAAACGACGGCCAGTGCACCTAGAAGGGGAAGTG | Forward | 6103 |
| **P-7R** | CAGGAAACAGCTATGACAGACAACCCATCTRGTCATTGGA (R=A/G) | Reverse | 7427 |
| **P-8F** | GTAAAACGACGGCCAGCCCATTAGTRTTCCCCTCTG (R=A/G) | Forward | 7067 |
| **P-8R** | CAGGAAACAGCTATGACTCCATTAATAATGGGATCCATT | Reverse | 8459 |
| **P-9F** | GTAAAACGACGGCCAGTGCCAGCAGACGTATTGAAG | Forward | 8029 |
| **P-9RF** | CAGGAAACAGCTATGACTTTATTATGTAGAACCCCTCATTGTG | Reverse | 9464 |
| **P-10F** | GTAAAACGACGGCCAGCAATGCAACATCCTCCATCA | Forward | 9053 |
| **P-10R** | CAGGAAACAGCTATGACGGTTGCATTGCAAACATTCTA | Reverse | 10381 |
| **P-11F** | GTAAAACGACGGCCAGCGTGAGTTTCGGTTGCCTA | Forward | 10033 |
| **P-11R** | CAGGAAACAGCTATGACGGGATCACCACCACCAAATA | Reverse | 11436 |
| **P-12F** | GTAAAACGACGGCCAGAGTGGGACCGTGGATAAACA | Forward | 11133 |
| **P-12R** | CAGGAAACAGCTATGACTGACTGTAAGGCGATGCAAA | Reverse | 12493 |
| **P-13F** | GTAAAACGACGGCCAGTGGACATCAAATATACWACAAGCA (W=A/T) | Forward | 12149 |
| **P-13R** | CAGGAAACAGCTATGACTTAACAACCCAAGGGCAAAC | Reverse | 13349 |
| **P-14F** | GTAAAACGACGGCCAGAAAAAGATTGGGGAGAGGGATA | Forward | 13010 |
| **P-14R** | CAGGAAACAGCTATGACTGCAYTTTCTTACATGCTTGC (Y=T/C) | Reverse | 14344 |
| **P-15F** | GTAAAACGACGGCCAGGGTGAAGGAGCAGGGAATTT | Forward | 14023 |
| **P-15R** | CAGGAAACAGCTATGACACGAGAAAAAAAGTGTCAAAAACT | Reverse | 15198 |

**Footnote:**

*Each forward primer had M13/pUC (-20) sequencing primer and **each** reverse primer had M13R-pUC(-26) sequencing primer attached at 5’end of primer.

**Primer binding positions are based on the genome sequence of HRSVA2 (Gene Bank accession number M74568).
