# Supplementary material for: Whole genome characterization of non-tissue culture adapted HRSV strains in severely infected children
Source: Virol J. 2011 Jul 28;8:372. doi: 10.1186/1743-422X-8-372 (PMC3166936; doi:10.1186/1743-422X-8-372)
Supplement: Additional file 2 — Figure S1: Amino acid sequence alignment and comparative analysis of glycoprotein between primary HRSVA strains and prototype cultured strains. The domain name with amino acid position is indicated above the sequence alignment. [file 1743-422X-8-372-S2.PDF]

Figure S1.

```
           1-37 Cytoplasmic                38-66  Transmembrane            67-298 Ectodomain
RSV-1      MSKNKDQRTAKTLERTWDTLNHLLFISSCLYKLNLKSVAQITLSILAMIISTSLIIAAIIFIASANHKVTPTTAIIQDATSQIKNTTPTYLTQNPQLGISPSNPSEITSQITTILASTTP
RSV-2      ........................................................................................................................
RSV-3      ........................................................................................................................
RSV-4      ........................................................................................................................
RSV-5      ........................................................................................................................
RSV-6      ........................................................................................................................
RSV-7      ........................................................................................................................
RSV-8      ........................................................................................................................
RSV-9      ........................................................................................................................
RSV-10     ........................................................................................................................
RSV-11     .....................................I..................................................................................
RSV-12     ....................................................................................................H...................
RSV-13     ........................................................................................................................
RSV-14     ........................................................................................................................
A2         ........................................................................................................................
RSS        .........T....K......................I................................L.............................F..L..T...T.........
Long       ..............K............G........I..................T.............L......................D......F..L......T.........
Line_19    ..............K......................I..................T.............L......................D......F..L......T.........

                                                  164-------------------176 Central conserved domain
                                                                        182--------------------------198 Heparin Binding site

RSV-1      GVKSTLQSTTVKTKNTTTTQTQPSKPTTKQRQNKPPTKPNNDFHFEVFNFVPCSICSNNPTCWAICKRIPNKKPGKKTTTKPTKKPTLKTTKKDLKPQTTKPKEVPTTKPTEKPTINTTK
RSV-2      ..........A.........I...............N...D..............................................I................A.S....D........
RSV-3      ....................................S........................................................EP......S..................
RSV-4      ....................................S.........................................................P......S..........E.......
RSV-5      ....................................S.........................................................P......S..........E.......
RSV-6      ....................................S.........................................................P......S..........E.......
RSV-7      ....................................S.........................................................P......S..........E.......
RSV-8      ....................................S.........................................................P......S..........E.......
RSV-9      ....................................S.........................................................P......S..........E.......
RSV-10     ....................................S.........................................................P......S..........E.......
RSV-11     ....................................S.........................................................P.........AF..............
RSV-12     .....R..............................N.........................................................P......S..........E.......
RSV-13     ....................................S.........................................................P......S........P.E.......
```

RSV-14 ....................................S...P......S..........E.......
A2 ....................................S...P......S..........E.......
RSS S..................KI...............N..I................................
Long ....N..P............................N..F........................E.......
Line_19 ....N..P............................N..F........................E.......

RSV-1 TNIITTLLTSNTTGNPELTSQEETFHSTSSGGNTSPSQIYTTSEYLSQPPSPSNITDQ
RSV-2 P..R.....NS....L.H......L.....E...........................
RSV-3 .................H...K..L...T.E..P..............SL....T.KW
RSV-4 .....................M........E.....K....................
RSV-5 ...............L.H...K.IL.....E...........................
RSV-6 .....................M.........H..F.A.....................
RSV-7 .....................M........E..S......S.................
RSV-8 .....................M........E...........................
RSV-9 .....................V..L.....E...........................
RSV-10 .....................K........E...........................
RSV-11 ...G..Q.A......L.Y...K..L.....E..P....V......P........T.NK
RSV-12 .....................M........E..P....V.........SL....T.KW
RSV-13 ...R...........Q.H...M..L...T.E..P....V.........SL....T.RW
RSV-14 ...K.............H...M..L...T.E..P....V.........SL....T.KW
A2 .....................M........E..P....VS.....P...S..P.TPR.
RSS ...R.....N.......H...KG.L.....D..P....V...............T.N.
Line_19 ...T.....N......K....M........E..L....VS....HP...S..P.T.R.
