# Supplementary material for: Whole genome characterization of non-tissue culture adapted HRSV strains in severely infected children
Source: Virol J. 2011 Jul 28;8:372. doi: 10.1186/1743-422X-8-372 (PMC3166936; doi:10.1186/1743-422X-8-372)
Supplement: Additional file 3 — Figure S2: Amino acid sequence alignment and comparative analysis of fusion protein between primary HRSVA strains and prototype cultured strains. The domain name with amino acid position is indicated above the sequence alignment. All the glycosylation sites are given in bold and underlined. [file 1743-422X-8-372-S3.PDF]

Figure S2. aa 1-22 Signal domain aa 27-529 Extracellular 109-110

```
         M-1
RSV-1    MELPILKTNAITTIFAAVTLCFASSQNITEEFYQSTCSAVSKGYLSALRTGWYTSVITIELSNIKENKCNGTDAKVKLIKQELDKYKNAVTELQLLMQSTPAANNRARRELPRFMNYTLN
RSV-2    ........................................................................................................................
RSV-3    ..............L.........................................................................................................
RSV-4    ..............LT...F....................................................................................................
RSV-5    .D..........A.L...S.....................................................................................................
RSV-6    ............A.L...S.....................................................................................................
RSV-7    ........................................................................................................................
RSV-8    ........................................................................................................................
RSV-9    ........................................................................................................................
RSV-10   ........................................................................................................................
RSV-11   .......I......L..I......................................................................................................
RSV-12   ..............L.........................................................................................................
RSV-13   .D............L.......................................................................................S.................
RSV-14   ..............L.........................................................................................................
A2       ...L...A......LT...F....G............................................................................PT.................
RSS      ............A.L...................T....................................................S..............T.................
Line_19  .......A......L....F.............................................K............M.........................................
```

136-137

```
RSV-1    NPKKTNVTLSKKRKRRFLVFLLGVGSAIASGIAVSKVLHLEGEVNKIRSALQSTNKAVVSLSNGVSVLTSKVLDLKNYIDKQLLPIVNKQSCSISNIETVIEFQQKNNRLLEITREFSVN
RSV-2    .T.NN.............G............................K...L....................................................................
RSV-3    .T.T..............G............................K...L....................................................................
RSV-4    .T.NN.............G............................K...L....................................................................
RSV-5    .T.NN.............G............................K...L....................................................................
RSV-6    .T.NN.............G............................K...L....................................................................
RSV-7    .T.NN.............G............................K...L....................................................................
RSV-8    .T.NN.............G............................K...L....................................................................
RSV-9    .T.NN.............G............................K...L....................................................................
RSV-10   .T.NN.............G............................K...L....................................................................
RSV-11   .T.N....V.........G............................K...L....................................................................
RSV-12   .T.T..............G............................K...L....................................................................
RSV-13   .T.N..............G............................K...L........FID.........................................................
RSV-14   .T.T..............G............................K...L....................................................................
A2       .A.............................V...............K...L.....L..............................................................
RSS      .T.N..............G............................K...L....................................................................
```

```
Long    .T................G............................K...L........................................R...........................
Line_19 .T................G............................K...L..................R.....................R...........................

RSV-1   AGVTTPVSTYMLTNSELLSLINDMPITNDQKKLMSNNVQIVRQQSYSIMSIIKEEVLAYVVQLPLYGVIDTPCWKLHTSPLCTTNTKEGSNICLTRTDRGWYCDNAGSVSFFPQAETCKV
RSV-2   ........................................................................................................................
RSV-3   ........................................................................................................................
RSV-4   ........................................................................................................................
RSV-5   ........................................................................................................................
RSV-6   ........................................................................................................................
RSV-7   ........................................................................................................................
RSV-8   ........................................................................................................................
RSV-9   ........................................................................................................................
RSV-10  ........................................................................................................................
RSV-11  ........................................................................................................................
RSV-12  ........................................................................................................................
RSV-13  ........................................................................................................................
RSV-14  ........................................................................................................................
A2      ........................................................................................................................
RSS     .................................................................................................................L......
Long    ........................................................................................................................
Line_19 ....................................................................................................................K...

RSV-1   QSNRVFCDTMNSLTLPSEVNLCNVDIFNPKYDCKIMTSKTDVSSSVITSLGAIVSCYGKTKCTASNKNRGIIKTFSNGCDYVSNKGVDTVSVGNTLYYVNKQEGKSLYVKGEPIINFYDP
RSV-2   ........................................................................................................................
RSV-3   ........................................................................................................................
RSV-4   ........................................................................................................................
RSV-5   ........................................................................................................................
RSV-6   ........................................................................................................................
RSV-7   ........................................................................................................................
RSV-8   ........................................................................................................................
RSV-9   ........................................................................................................................
RSV-10  ........................................................................................................................
RSV-11  ........................................................................................................................
RSV-12  .......................I................................................................................................
RSV-13  .......................I................................................................................................
RSV-14  ........................................................................................................................
A2      ..................I...................................................................M.................................
```

```
RSS      .......................I................................................................................................
Long     ........................................................................................................................
Line_19  ..........Y.............................................................................................................

                                                     530-550 Transmembrane     551-574 Cytosolic
                                                                  |                    |
                                                                  v                    v
RSV-1    LVFPSDEFDASISQVNEKINQSLAFIRKSDELLHNVNVGKSTTNIMITTIIIVIIVILLLLIAVGLFLYCKARSTPVTLIKDQLSGINNIAFSN
RSV-2    ...............................................................................S..............
RSV-3    ...........................................................S......L...........VS..............
RSV-4    ..................................................................L............S..............
RSV-5    ...............................................................................S..............
RSV-6    ..................................................................L............S..............
RSV-7    .....................................A.....................S......L............S..............
RSV-8    ..............................................................................V.D.Y.......S.IY
RSV-9    ...............................................................................S..............
RSV-10   ..................................................................L...........V.N.............
RSV-11   ...........................................................S......L............S..............
RSV-12   .....................................A.....................S......L............S.............S
RSV-13   ...........................................................S......L............S..............
RSV-14   ...........................................................S......L............S.............S
A2       .....................................A.....................S......L............S..............
RSS      .....................................A....I................S......L............S..............
Long     ..................................H..A.....................S......L............S..............
Line_19  .....................................A.....................S......L.........I..S..............
```
