# Supplementary material for: Whole genome characterization of non-tissue culture adapted HRSV strains in severely infected children
Source: Virol J. 2011 Jul 28;8:372. doi: 10.1186/1743-422X-8-372 (PMC3166936; doi:10.1186/1743-422X-8-372)
Supplement: Additional file 4 — Figure S3: Amino acid alignment and comparative analysis of L-protein between primary HRSVA strains and prototype cultured strains. [file 1743-422X-8-372-S4.PDF]

Figure S 3

```
RSV-1    MDPIINGNSANVYLTDSYLKGVISFSECNALGSYIFNGPYLKNDYTNLISRQNPLIEHINLKKLNITQSLISKYHKGEIKIEEPTYFQSLLMTYKSMTSSEQIATTNLLKKIIRRAIEIS
RSV-2    .......................................................................................................T................
RSV-3    .......................................................................................................T................
RSV-4    .......................................................................................................T................
RSV-5    ........................................................................................................................
RSV-6    .......................................................................................................T................
RSV-7    .......................................................................................................S................
RSV-8    .......................................................................................................T................
RSV-9    .......................................................................................................T................
RSV-10   .......................................................................................................T................
RSV-11   ..............................................S........................................................T................
RSV-12   .....S.................................................................................................T................
RSV-13   .....S.................................................................................................T................
RSV-14   .......................................................................................................T................
A2       ..........................................................M.....................L.......................................
RSS      ......................................................................M............................L...T................
LONG     ..........................................................M.....................L..................L....................
Line_19  ..........................................................M.....................L..................L....................

RSV-1    DVKVYAILNKLGLKEKDKIKSNNGQDENNSVITTIIKDDILLAVKDNQSHLKAGKNHSTKQKDTIKTTLLKKLMCSMQHPPSWLIHWFNLYTKLNNILTQYRSNEVKNHGFILIDNHTLN
RSV-2    ........................................................................................................................
RSV-3    ...........................D...................................................................S.......S................
RSV-4    ........................................................................................................................
RSV-5    ........................................................................................................................
RSV-6    ........................................................................................................................
RSV-7    ........................................................................................................................
RSV-8    ........................................................................................................................
RSV-9    ........................................................................................................................
RSV-10   ....................F......Y.........................D..................................................................
RSV-11   ....................F.S....D.........................D.................................................S................
RSV-12   ...........................D.........................D.S.......................................S.......S................
RSV-13   ...........................D.........................D.S.......................................S.......S................
RSV-14   ........................................................................................................................
A2       ...........................D.............S...........D.........................................................T....Q..S
RSS      ...........................D.........................V.................................................S................
LONG     ...........................D.............S...........D..............................................................Q..S
Line_19  ...........................D.............S...........D..............................................................Q..S

RSV-1    GFQFILNQYGCIVYHKDLKRITVTTYNQFLTWKDISLSRLNVCLITWISNCLNTLNKSLGLRCGFNNVILTQLFLYGDCILKLFHNEGFYIIKEVEGFIMSLILNITEEDQFRKRFYNSM
RSV-2    ................E.......................................................................................................
RSV-3    ................E.........................................................H.............................................
RSV-4    ................E.............................................W.........................................................
RSV-5    ........................................................................................................................
RSV-6    ................E.......................................................................................................
RSV-7    ........................................................................................................................
RSV-8    ................E.......................................................................................................
RSV-9    ................E.......................................................................................................
RSV-10   ......S.........E.......................................................................................................
RSV-11   ......S.........E.......................................................................................................
RSV-12   ................E.....M...................................................H.............................................
RSV-13   ................E.....M...................................................H................................K............
RSV-14   ................E.....M...................................................H.............................................
A2       ................E.......................................................................................................
RSS      ................E................N......................................................................................
```

```
LONG     ................E.......................................................................................................
Line_19  ................E.......................................................................................................

RSV-1    LNNITDAANKAQKNLLSRVCHTLLDKTVSDNIINGRWIILLSKFLKLIKLAGDNNLNNLSELYFLFRIFGHPMVDERQAMDAVKVNCNETKFYLLSSLSMLRGAFIYRIIKGFVNNYNRW
RSV-2    ........................................................................................................................
RSV-3    ........................................................................................................................
RSV-4    ....................................................................................I...................................
RSV-5    ........................................................................................................................
RSV-6    ....................................................................................I...................................
RSV-7    ........................................................................................................................
RSV-8    ....................................................................................I...................................
RSV-9    ........................................................................................................................
RSV-10   ....................................................................................I...................................
RSV-11   ........................................................................................................................
RSV-12   ........................................................................................................................
RSV-13   ........................................................................................................................
RSV-14   ........................................................................................................................
A2       ....................................................................................I...................................
RSS      ........................................................................................................................
LONG     ........................................................................................................................
Line_19  ........................................................................................................................

RSV-1    PTLRNAIVLPLRWLTYYKLNTYPSLLELTERDLIVLSGLRFYREFRLPKKVDLEMIINDKAISPPKNLIWTSFPRNYMPSHIQNYIEHEKLKFSESDKSRRVLEYYLRDNKFNECDLYNC
RSV-2    ........................................................................................................................
RSV-3    ........................................................................................................................
RSV-4    ........................................................................................................................
RSV-5    ...............................................................................P........................................
RSV-6    ........................................................................................................................
RSV-7    ........................................................................................................................
RSV-8    ........................................................................................................................
RSV-9    ..............................I........................M................................................................
RSV-10   ........................................................................................................................
RSV-11   ........................................................................................................................
RSV-12   ........................................................................................................................
RSV-13   ........................................................................................................................
RSV-14   ........................................................................................................................
A2       ........................................................................................................................
RSS      ........................................................................................................................
LONG     ........................................................................................................................
Line_19  ........................................................................................................................

RSV-1    VVNQSYLNNPNHVVSLTGKERELSVGRMFAMQPGMFRQVQILAEKMIAENILQFFPESLTRYGDLELQKILELKAGISNKSNRYNDNYNNYISKCSIITDLSKFNQAFRYETSCICSDVL
RSV-2    ........................................................................................................................
RSV-3    ........................................................................................................................
RSV-4    ........................................................................................................................
RSV-5    ........................................................................................................................
RSV-6    ........................................................................................................................
RSV-7    ........................................................................................................................
RSV-8    ........................................................................................................................
RSV-9    ........................................................................................................................
RSV-10   ........................................................................................................................
RSV-11   ........................................................................................................................
RSV-12   ........................................................................................................................
RSV-13   ........................................................................................................................
RSV-14   ........................................................................................................................
A2       ........................................................................................................................
RSS      ........................................................................................................................
```

```
LONG    ........................................................................................................................
Line_19 ........................................................................................................................

RSV-1   DELHGVQSLFSWLHLTIPHVTIICTYRHAPPYIRDHIVDLNNVDEQSGLYRYHMGGIEGWCQKLWTIEAISLLDLISLKGKFSITALINGDNQSIDISKPVRLMEGQTHAQADYLLALNS
RSV-2   ........................................................................................................................
RSV-3   ........................................................................................................................
RSV-4   ........................................................................................................................
RSV-5   ........................................................................................................................
RSV-6   ........................................................................................................................
RSV-7   ......................KM................................................................................................
RSV-8   ....................................................Q...................................................................
RSV-9   ........................................................................................................................
RSV-10  ........................................................................................................................
RSV-11  ........................................................................................................................
RSV-12  .................................K.P....................................................................................
RSV-13  ........II.......................K.P....................T...............................................................
RSV-14  ........................................................................................................................
A2      .................................G..................................................................I...................
RSS     ..........F....A........................................................................................................
LONG    ........................................................................................................................
Line_19 ........................................................................................................................

RSV-1   LKLLYKEYAGIGHKLKGTETYISRDMQFMSKTIQHNGVYYPASIKKVLRVGPWINTILDDFKVSLESIGSLTQELEYRGESLLCSLIFRNVWLYNQIALQLKNHALCNNKLYLDILRVLK
RSV-2   ....................................................................................................................K...
RSV-3   ....................................................................................................................K...
RSV-4   ....................................................................................................................K...
RSV-5   ....................................................................................................................K...
RSV-6   ........................................................................................................................
RSV-7   ........................................................................................................................
RSV-8   ....................................................................................................................K...
RSV-9   ....................................................................................................................K...
RSV-10  ....................................................................................................................K...
RSV-11  ....................................................................................................................K...
RSV-12  ....................................................................................................................K...
RSV-13  ....................................................................................................................K...
RSV-14  ....................................................................................................................K...
A2      ....................................................................................................................K...
RSS     ....................................................................................................................K...
LONG    ....................................................................................................................K...
Line_19 ....................................................................................................................K...

RSV-1   HLKTFFNLDNIDTALTLYMNLPMLFGGGDPNLLYRSFYRRTPDFLTEAIVHSVFILSYYTNHDLKDKLQDLSDDRLNKFLTCIITFDKNPNAEFVTLMRDPQALGSERQAKITSEINRLA
RSV-2   ........................................................................................................................
RSV-3   ........................................................................................................................
RSV-4   ........................................................................................................................
RSV-5   ........................................................................................................................
RSV-6   ........................................................................................................................
RSV-7   ........................................................................................................................
RSV-8   ........................................................................................................................
RSV-9   ........................................................................................................................
RSV-10  ...S..................................................V.................................................................
RSV-11  ...S..................................................V.................................................................
RSV-12  ........................................................................................................................
RSV-13  ........................................................................................................................
RSV-14  ........................................................................................................................
A2      ........................................................................................................................
RSS     ........................................................................................................................
```

```
LONG     ........................................................................................................................
Line_19  ........................................................................................................................

RSV-1    VTEVLSTAPNKIFSKSAQHYTTTEIDLNDIMQNIEPTYPHGLRVVYESLPFYKAEKIVNLISGTKSITNILEKTSAIDLTDIDRATEMMRKNITLLIRIFPLDCNRDKREILSMENLSIT
RSV-2    ........................................................................................................................
RSV-3    ..........................................................................................................LL....................
RSV-4    ...................................................................................................L....................
RSV-5    ........................................................................................................................
RSV-6    ........................................................................................................................
RSV-7    ........................................................................................................................
RSV-8    ...................................................................................................L....................
RSV-9    ........................................................................................................................
RSV-10   ...I.................................................................................V..................................
RSV-11   ...I.................................................................................V..................................
RSV-12   ..................................................................................................LL....................
RSV-13   ..................................................................................................LL....................
RSV-14   ..................................................................................................LL....................
A2       ...................................................................................................L....................
RSS      ........................................................................................................................
LONG     ...................................................................................................L....................
Line_19  ...................................................................................................L....................

RSV-1    ELSKYVRERSWSLSNIVGVTSPSIMYTMDIKYTTSTIASGIIIEKYNVNSLTRGERGPTKPWVGSSTQEKKTMPVYNRQVLTKKQRDQIDLLAKLDWVYASIDNKDEFMEELSIGTLGLT
RSV-2    ........................................................................................................................
RSV-3    ........................................................................................................................
RSV-4    .....................................S..................................................................................
RSV-5    ........................................................................................................................
RSV-6    ........................................................................................................................
RSV-7    ........................................................................................................................
RSV-8    .....................................S..................................................................................
RSV-9    ........................................................................................................................
RSV-10   ........................................................................................................................
RSV-11   ........................................................................................................................
RSV-12   ........................................................................................................................
RSV-13   ........................................................................................................................
RSV-14   ........................................................................................................................
A2       .....................................S..................................................................................
RSS      ...................................................................................................................I....
LONG     ........................................................................................................................
Line_19  ........................................................................................................................

RSV-1    YEKAKKLFPQYLSVNYLHRLTVSSRPCEFPASIPAYRTTNYHFDTSPINRILTEKYGDEDIDIVFQNCISFGLSLMSVVEQFTNVCPNRIILIPKLNEIHLMKPPIFTGDVDIHKLKQVI
RSV-2    ........................................................................................................................
RSV-3    ........................................................................................................................
RSV-4    ........................................................................................................................
RSV-5    ........................................................................................................................
RSV-6    ........................................................................................................................
RSV-7    ........................................................................................................................
RSV-8    ........................................................................................................................
RSV-9    ..................................................V.....................................................................
RSV-10   ........................................................................................................................
RSV-11   ........................................................................................................................
RSV-12   ....................................................................................A...................................
RSV-13   ........................................................................................................................
RSV-14   ........................................................................................................................
A2       ........................................................................................................................
RSS      ........................................................................................................................
```

```
LONG    ........................................................................................................................
Line_19 ........................................................................................................................

RSV-1   QKQHMFLPDKISLTQYVELFLSNKTLKSGSHVNSNLILAHKISDYFHNTYILSTNLAGHWILIIQLMKDSKGIFEKDWGEGYITDHMFINLKVFFNAYKTYLLCFHKGYGRAKLECDMNT
RSV-2   ........................................................................................................................
RSV-3   ..............................N.........................................................................................
RSV-4   ..............................................................................................................K.........
RSV-5   ........................................................................................................................
RSV-6   ..............................................................................................................K.........
RSV-7   ..............................................................................................................K.........
RSV-8   ........................................................................................................................
RSV-9   ........................................................................................................................
RSV-10  ........................................................................................................................
RSV-11  ........................................................................................................................
RSV-12  ..............................N..............................................................................S..........
RSV-13  ........................................................................................................................
RSV-14  .................................................................H......................................................
A2      ..............................................................................................................K.........
RSS     ........................................................................................................................
LONG    ..............................................................................................................K.........
Line_19 ..............................................................................................................K.........

RSV-1   SDLLCVLELIDSSYWKSMSKVFLEQKVIKYILSQDASLHRVKGCHSFKLWFLKRLNVAEFTVCPWVVNIDYHPTHMKAILTYIDLVRMGLINIDRIYIKNNHKFNDEFYTSNLFYINYNF
RSV-2   ....................................................................................................K...................
RSV-3   ....................................................................................................K...................
RSV-4   ................................................................................................H...K...................
RSV-5   ....................................................................................................K...................
RSV-6   ................................................................................................H...K...................
RSV-7   ........................................................................................................................
RSV-8   ....................................................................................................K...................
RSV-9   ...............................................................................................M....K...................
RSV-10  ......................................................................................G.............K...................
RSV-11  ....................................................................................................K...................
RSV-12  ...................................S................................................................K...................
RSV-13  ....................................................................................................K...................
RSV-14  ....................................................................................................K....E....Y.........
A2      ................................................................................................H...K...................
RSS     ..............................................................................................K.....K...................
LONG    ................................................................................................H...K...................
Line_19 ................................................................................................H...K...................

RSV-1   SDNTHLLTKHIRIANSELENNYNKLYHPTPETLENILTNPVKCDDKKTLNDYCIGKNVDSIMLPLLSNKKLIKSSTTIRTNYSKQDLYNLFPTVVIDKIIDHSGNTAKSNQLYTTTSHQI
RSV-2   ............................................................................M...........................................
RSV-3   ...................S......................NN....................S...........M......R....................................
RSV-4   .....................................A..I.SN...............................AM...............M....R......................
RSV-5   ..................................................................P.....................................................
RSV-6   .....................................A..I.SN...............................AM...............M....R......................
RSV-7   ........................................................................................................................
RSV-8   ............................................................................M...........................................
RSV-9   .........................................................I..................M.............................S.............
RSV-10  ............V.............................SN....................F....R......M......R....................................
RSV-11  ............V.............................SN....................F....R......M......R....................................
RSV-12  ...................C......................SN....................S...........M......R....................................
RSV-13  ...................S......................SN.....S.............TS...........M......R....................................
RSV-14  ..................................................................P.....................................................
A2      .....................................A..I.SN...............................AM...............M....R......................
RSS     ...........................................N..............................P.M...........................................
```

```
LONG     .....................................A..I.SN...........................V...AM....................R......................
Line_19  .....................................A..I.SN...........................V...AM....................R......................

RSV-1    SLVHNSTSLYCMLPWHHINRFNFVFSSTGCKISIEYILKDLKIKDPSCIAFIGEGAGNLLLRTVVELHPDIRYIYRSLKDCNDHSLPIEFLRLYNGHINIDYGENLTIPATDATNNIHWS
RSV-2    ..............................................N.........................................................................
RSV-3    ..............................................N.........................................................................
RSV-4    ..............................................N.........................................................................
RSV-5    ........................................................................................................................
RSV-6    ..............................................N.........................................................................
RSV-7    .............................................T..........................................................................
RSV-8    ..............................................N.........................................................................
RSV-9    ..............................................N.........................................................................
RSV-10   ..............................................N.........................................................................
RSV-11   ..............................................N.........................................................................
RSV-12   ..............................................N.........................................................................
RSV-13   ..............................................N.........................................................................
RSV-14   ........................................................................................................................
A2       ..............................................N.........................................................................
RSS      P........................................I....N.........................................................................
LONG     ..............................................N.........................................................................
Line_19  ..............................................K.........................................................................

RSV-1    YLHIKFAEPISLFVCDAELPVTVNWSKIIIEWSKHVRKCKYCSSVNKCTLIVKYHAQDDIDFKLDNITILKTYVCLGSKLKGSEVYLVLTIGPANVFPVFNVVQNAKLILSRTKNFIMPK
RSV-2    ........................................................................................................................
RSV-3    .............................M..........................................................................................
RSV-4    ........................................................................................................................
RSV-5    ........................................................................................................................
RSV-6    ........................................................................................................................
RSV-7    ........................................................................................................................
RSV-8    ........................................................................................................................
RSV-9    ........................................................................................................................
RSV-10   ....................AI..................................................................................................
RSV-11   ....................AI..................................................................................................
RSV-12   ............................................................N...........................................................
RSV-13   ............................................................N...........................................................
RSV-14   ........................................................................................................................
A2       ...................S............................M..............................................I........................
RSS      ........................................................................................................................
LONG     ...............................................................................................I........................
Line_19  ...............................................................................................I........................

RSV-1    KADKESIDANIKSLIPFLCYPITKKGINTALSKLKSVVSGDILSYSIAGRNEVFSNKLINHKHMNILKWFNHVLNFRSTELNYNHLYMVESTYPYLSELLNSLTTNELKKLIKITGSLLY
RSV-2    ........................................................................................................................
RSV-3    ..............................................................................................H.........................
RSV-4    ........................................................................................................................
RSV-5    ........................................................................................................................
RSV-6    ........................................................................................................................
RSV-7    ........................................................................................................................
RSV-8    ........................................................................................................................
RSV-9    ........................................................................................................................
RSV-10   ........................................................................................................................
RSV-11   ........................................................................................................................
RSV-12   ..............................................................................................H.........................
RSV-13   ..............................................................................................H.........................
RSV-14   ........................................................................................................................
A2       ........................................................................................................................
RSS      ........................................................................................................................
```

```
LONG    ........................................................................................................................
Line_19 ........................................................................................................................

RSV-1   NFHNE
RSV-2   .....
RSV-3   ..N..
RSV-4   .....
RSV-5   .....
RSV-6   .....
RSV-7   .....
RSV-8   .....
RSV-9   .....
RSV-10  .....
RSV-11  .....
RSV-12  ..N..
RSV-13  ..N..
RSV-14  .....
A2      .....
RSS     .....
LONG    .....
Line_19 .....
```
