# Supplementary material for: Whole genome characterization of non-tissue culture adapted HRSV strains in severely infected children
Source: Virol J. 2011 Jul 28;8:372. doi: 10.1186/1743-422X-8-372 (PMC3166936; doi:10.1186/1743-422X-8-372)
Supplement: Additional file 5 — Figure S4: Amino acid alignment and comparative analysis of M2-1 protein between primary HRSVA strains and prototype cultured strains. [file 1743-422X-8-372-S5.PDF]

Figure S4.

```
RSV-1      MSRRNPCKFEIRGHCLNGKRCHFSHNYFEWPPHALLVRQNFMLNRILKSMDKSIDTLSEISGAAELDRTEEYALGVVGVLESYIGSINNITKQSACVAMSKLLTELNSDDIKKLRDNEEP
RSV-2      ...........................................................................................................G............
RSV-3      .......................................................................................................................L
RSV-4      .......................................................................................................................L
RSV-5      ...........................................................................................................G............
RSV-6      .......................................................................................................................L
RSV-7      .......................................................................................................................L
RSV-8      ....................................................................................................................T...
RSV-9      ........................................................S..............................................................L
RSV-10     ....................................................................................................................T...
RSV-11     ....................................................................................................................T...
RSV-12     ..................R................................R..................................L.............................S...
RSV-13     ..................R................................R..................................L.............................S...
RSV-14     ..................R................................R..................................L.............................S...
A2         .......................................................................................................................L
RSS        ........................................................................................................................
LONG       .......................................................................................................................L
Line_19    .......................................................................................................................L

RSV-1      NSPKIRVYNTVISYIESNRKNNKQTIHLLKRLPADVLKKTIKNTLDIHKSITINNPKESTVNDTNDHAKNNDTT
RSV-2      ..........................................................................
RSV-3      .............................................................S...V........
RSV-4      .............................................................S............
RSV-5      ..........................................................................
RSV-6      .............................................................S............
RSV-7      .............................................................S............
RSV-8      ..............................TV..........................................
RSV-9      ....V.....................................................................
RSV-10     ........................................M....................S...Y........
RSV-11     .............................................................S..DY........
RSV-12     ....V......T.................................................S............
RSV-13     ....V......T...................V.............................S............
RSV-14     ....V......T.................................................S............
A2         .............................................................S............
RSS        ..........................................T..................S.I..........
LONG       ..........................................................L..S............
Line_19    ..........................................................L..S............
```
