# Supplementary material for: Whole genome characterization of non-tissue culture adapted HRSV strains in severely infected children
Source: Virol J. 2011 Jul 28;8:372. doi: 10.1186/1743-422X-8-372 (PMC3166936; doi:10.1186/1743-422X-8-372)
Supplement: Additional file 6 — Figure S5: Amino acid alignment and comparative analysis of M2-2 protein between primary HRSVA strains and prototype cultured strains. [file 1743-422X-8-372-S6.PDF]

Figure S5.

```
RSV-1   TTMPKIMILPDKYPCSINSILITSSCRVTMYNQKNTLYFNQNNQNNHTYSPNQPFNEIHWTSQDLIDAIQNFLQHLGITDDIYTIYILVS
RSV-2   ..........................................................................................
RSV-3   MS...............T......R..................P...M.....T..............T.....................
RSV-4   M................T......R......................M..........................................
RSV-5   ..........................................................................................
RSV-6   M................T......R..................P...M..........................................
RSV-7   M.......................R..................P..................................I...........
RSV-8   ..........................................................................................
RSV-9   ..........................................................................................
RSV-10  ...............................................I....................T.........I...........
RSV-11  .........................................K.....I....................T.........I...........
RSV-12  ........................N.............I........I..Q.................T.....................
RSV-13  ........................N.............I........I..Q.................T.....................
RSV-14  ........................N.............I........I..Q.......................................
RSVA2   M................T......R..................P...M.....T.........E...T..........IE..........
RSS     ...............................................I..........................................
LONG    M................T......R.......R..........P...M.....T.............T.........VIE..........
Line_19 M................T......R.......R..........P...M.....T.............T.........VIE..........
```
